# Conservative Management of Pulpitis in a Mature Permanent Molar Using Partial Pulpotomy: A Case Report

**DOI:** 10.7759/cureus.108010

**Published:** 2026-04-30

**Authors:** Alaa Eddin O Alostwani

**Affiliations:** 1 Dentistry, Medici Dental Center, Doha, QAT

**Keywords:** bioceramic materials, irreversible pulpitis, mature permanent teeth, minimally invasive dentistry, partial pulpotomy, pulp preservation, vital pulp therapy

## Abstract

Vital pulp therapy (VPT) has emerged as a conservative alternative to conventional root canal therapy (RCT) for the management of teeth with pulp exposure. Advances in bioactive materials have expanded its indications, allowing its application in mature permanent teeth presenting with symptoms traditionally associated with irreversible pulpitis. A 36-year-old female presented with severe pain in the right mandibular first molar. A partial pulpotomy using a bioceramic material was performed, followed by composite restoration. The patient became asymptomatic shortly after treatment. At 11 months, clinical and radiographic outcomes were favourable, and minor occlusal discomfort was resolved following occlusal adjustment. Partial pulpotomy using bioceramic materials may represent a predictable and minimally invasive treatment option for mature permanent teeth diagnosed with symptomatic pulpitis. Proper case selection, strict isolation, effective coronal sealing, and occlusal evaluation are essential for favourable outcomes.

## Introduction

Preservation of pulpal vitality is a central objective in modern restorative and endodontic dentistry. Traditionally, teeth presenting with symptoms suggestive of irreversible pulpitis were treated with conventional root canal therapy (RCT). However, recent advances in pulp biology have led to a paradigm shift toward more conservative management strategies [1,2].

Vital pulp therapy (VPT) includes procedures aimed at maintaining pulpal vitality by removing infected tissue while preserving healthy pulp. These include indirect pulp capping, direct pulp capping, partial pulpotomy, and full pulpotomy [3]. Among these, partial pulpotomy has gained increasing attention due to its minimally invasive nature and favourable outcomes [4].

Successful outcomes of VPT depend on strict case selection and adherence to key clinical principles. These include effective isolation, aseptic technique, and accurate assessment of pulpal status prior to treatment [3]. An important intraoperative indicator of pulpal health is the ability to achieve hemostasis within a short period, as prolonged or uncontrolled bleeding may reflect deeper pulpal inflammation and influence treatment prognosis [2,5].

In addition, the use of magnification enhances visualization of the pulp tissue, allowing improved assessment of tissue characteristics, including the texture of the exposed pulp, its degree of attachment to the surrounding dentinal walls, and the extent of inflammation. This facilitates more precise removal of inflamed tissue and contributes to improved clinical outcomes [3].

Historically, partial pulpotomy was mainly recommended for traumatic exposures in immature teeth. However, growing evidence suggests that pulpal inflammation in cases diagnosed as irreversible pulpitis may be localized to the coronal pulp, allowing preservation of the remaining pulp tissue [2,5]. The development of calcium silicate-based bioceramic materials has significantly improved the success of VPT due to their biocompatibility, sealing properties, and ability to stimulate dentin bridge formation [6,7]. Recent systematic reviews and clinical studies have demonstrated high success rates of partial pulpotomy in mature permanent teeth when appropriate case selection and clinical protocols are followed [8-10].

The aim of this case report is to describe the successful conservative management of symptomatic pulpitis using partial pulpotomy with a bioceramic material.

## Case presentation

A 36-year-old medically fit female patient, with no relevant medical history and not taking any medications, presented with severe pain related to the right mandibular first molar (tooth 46). The patient reported pain during mastication for approximately two weeks, accompanied by episodes of severe spontaneous pain lasting three to five minutes and recurring approximately every hour. Additionally, the patient described a sensation of numbness on the right side of the mouth.

Clinical examination

Intraoral examination revealed deep recurrent caries extending toward the pulp chamber. Cold testing using Endo-Ice (-50°C; Coltene/Whaledent Inc., Cuyahoga Falls, OH, USA) produced an exaggerated and prolonged response lasting approximately three minutes. Percussion testing was positive, indicating periodontal ligament (PDL) involvement, while palpation testing was negative. No abnormal mobility was detected.

Radiographic findings

**Figure 1:**
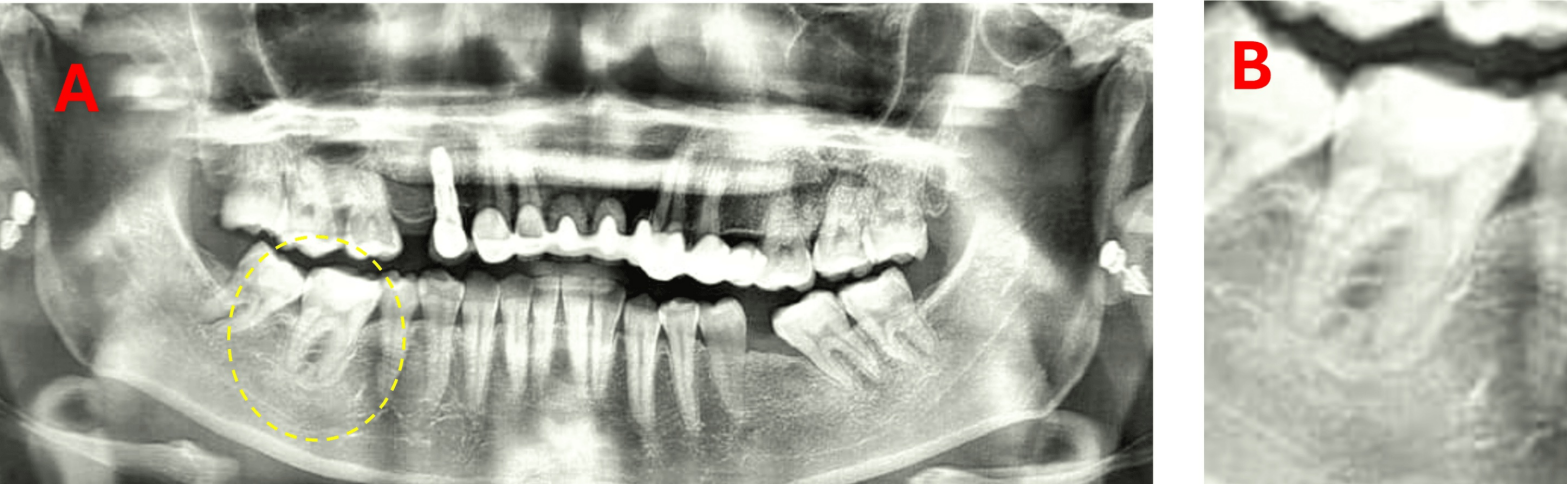
Preoperative radiographic findings. (A) Orthopantomogram showing tooth 46 (circled) with deep recurrent caries and periodontal ligament (PDL) widening. (B) Magnified view of the affected tooth.

Radiographic examination (orthopantomogram) demonstrated deep recurrent caries approaching the pulp chamber, along with widening of the PDL space around both mesial and distal roots. A localized area of increased radiopacity adjacent to the mesial root was also observed (Figure 1).

Treatment procedure

A conservative approach was selected to preserve pulpal vitality. The procedure was performed under rubber dam isolation following disinfection of the operative field.

Caries removal was carried out using a high-speed handpiece with a sterile diamond bur under copious water irrigation. Upon completion of caries removal, pulp exposure occurred. The procedure was performed under magnification using dental loupes (×3; Univet, Rezzato, Italy), allowing enhanced visualization and precise tissue management.

A partial pulpotomy was performed by removing a small portion of the coronal pulp using a sterile diamond bur under continuous water irrigation. The exposed pulp tissue appeared vital. Hemostasis was achieved within approximately three minutes using gentle irrigation with sterile saline to remove debris, followed by application of 2% sodium hypochlorite.

**Figure 2:**
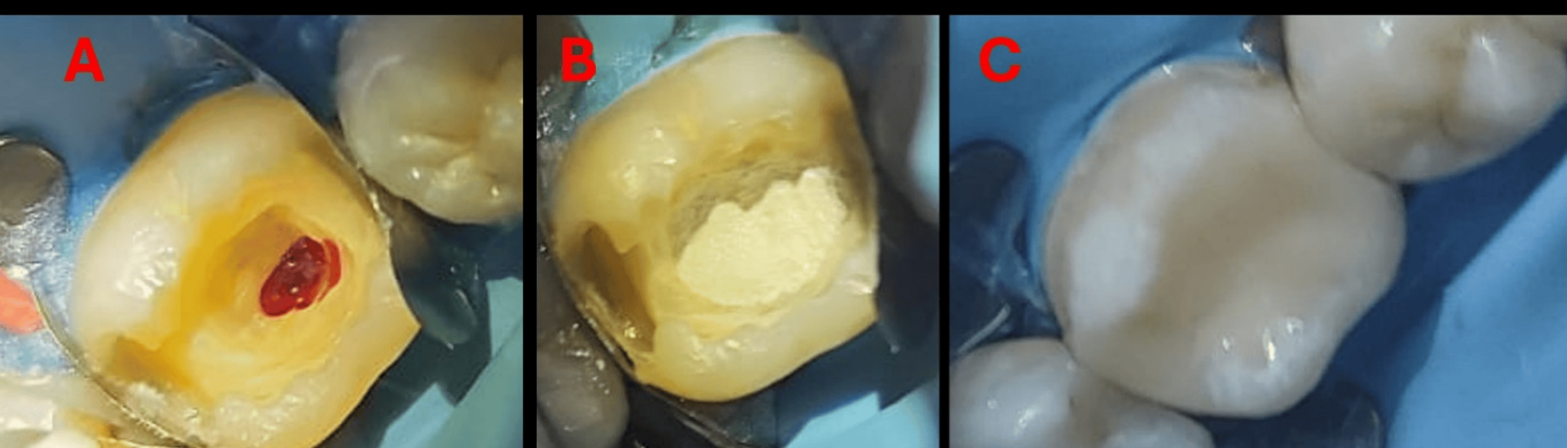
Clinical sequence. (A) Partial pulpotomy. (B) Bioceramic application. (C) Final restoration.

A premixed calcium silicate-based bioceramic pulp-capping material (Cumedent GmbH, Germany) was placed over the clinically assessed remaining vital pulp tissue. A base layer of TheraBase (BISCO Inc., Schaumburg, IL, USA) was then applied, followed by restoration using Tokuyama Estelite Posterior composite resin (shade A2; Tokuyama Dental Corp., Tokyo, Japan) to ensure an adequate coronal seal (Figure 2).

Postoperative outcome

No postoperative medications were prescribed. One day after treatment, the patient reported complete relief of symptoms and regained normal masticatory function.

Follow-up evaluation

**Figure 3:**
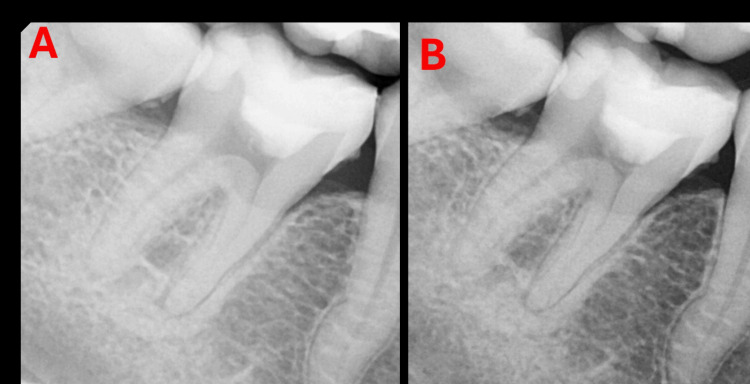
Radiographic follow-up. (A) Immediate postoperative radiograph. (B) Eleven-month follow-up showing healing.

At 11 months, radiographic evaluation demonstrated a reduction in the previously observed PDL widening, indicating ongoing healing and periodontal recovery. The surrounding bone appeared healthy, with no signs of periapical pathology. Additionally, slight calcification was observed within the pulp chamber, more prominently localized at the pulpotomy site (Figure 3).

Clinical findings included no mobility, negative percussion, and negative palpation. No discoloration of the treated tooth was observed at 11 months. However, the patient reported mild discomfort during mastication, particularly during the chewing of hard foods. Further clinical evaluation revealed slight occlusal interference. Following occlusal adjustment, the patient experienced immediate relief, and all symptoms resolved completely.

## Discussion

VPT has gained increasing acceptance as a biologically based approach for managing inflamed pulp tissue. Traditionally, symptomatic pulpitis was considered irreversible; however, current evidence suggests that inflammation may often be localized to the superficial coronal pulp, allowing conservative treatment and preservation of pulp vitality [2,5].

Partial pulpotomy specifically removes the inflamed superficial pulp while preserving the remaining healthy tissue, maintaining pulp vitality and physiological function. Clinical studies have demonstrated high success rates exceeding 85-90% in mature permanent teeth when strict clinical protocols are followed [8-10].

The use of calcium silicate-based bioceramic materials has significantly improved outcomes due to their bioactivity and sealing ability. These materials promote reparative dentin formation, reduce bacterial microleakage, and enhance pulpal healing [6,7]. Their composition, primarily consisting of calcium silicates, facilitates the release of calcium ions and promotes hydroxyapatite formation at the material-dentin interface, supporting dentin bridge formation and providing an effective biological seal [6,7].

In the present case, the tooth presented with clinical features suggestive of symptomatic irreversible pulpitis; however, it is important to recognize that clinical diagnostic terminology does not always precisely reflect the histological status of the pulp [2,5]. The favourable response observed following partial pulpotomy suggests that the inflammatory process may have been limited to the coronal pulp, allowing preservation of the remaining vital tissue.

The decision to perform partial pulpotomy instead of full pulpotomy or root canal treatment was based on intraoperative findings, including the presence of vital pulp tissue and the ability to achieve hemostasis within approximately three minutes, which is considered a favourable prognostic indicator of limited pulpal inflammation [2,8]. At 11 months, radiographs demonstrated a reduction in PDL widening and slight calcification within the pulp chamber, more prominently localized at the site of the partial pulpotomy, suggesting reparative dentin formation and ongoing healing, consistent with previous reports on VPT outcomes [8-11]. However, such calcific changes should be interpreted with caution and monitored over time, as excessive or diffuse calcification may complicate future endodontic treatment if required [7].

A localized area of increased radiopacity adjacent to the mesial root was observed preoperatively. While this finding may be suggestive of condensing osteitis, the absence of consistent clinical signs of pulp necrosis and the favourable intraoperative findings supported a conservative approach in this case. This emphasizes the importance of correlating radiographic findings with clinical examination rather than relying solely on radiographic interpretation [2,9]. Additionally, no discoloration was observed at 11 months, which is clinically relevant as some calcium silicate-based materials have been associated with esthetic concerns [7].

Importantly, this case also illustrates a conservative restorative approach. No full-coverage crown was placed; instead, the tooth was restored using direct posterior composite resin. This preserved tooth structure while achieving an adequate coronal seal, aligning with minimally invasive dentistry principles and current recommendations for VPT in mature permanent teeth [3,12].

Finally, mild postoperative discomfort was observed during mastication, which was related to slight occlusal interference rather than residual pulpal inflammation. Occlusal discrepancies can exert stress on the PDL and may mimic pulpal symptoms, emphasizing the importance of careful occlusal assessment during follow-up [13].

This case presents several limitations. The use of orthopantomographic imaging without a preoperative periapical radiograph limits the accuracy of assessing periapical status and subtle PDL changes, as such imaging may lack sufficient resolution and detail. Additionally, the follow-up period of 11 months is relatively short for evaluating long-term outcomes. Therefore, the findings of this case should be interpreted with caution, and further long-term clinical studies are required to support broader clinical application. Overall, while the favourable outcome observed in this case supports the potential of partial pulpotomy in selected cases, conclusions regarding its generalizability should be made cautiously due to the inherent limitations of a single case report [8-10].

## Conclusions

Partial pulpotomy with bioceramic materials may be considered a minimally invasive treatment option in carefully selected cases of symptomatic pulpitis in mature permanent teeth. Clinical success appears to depend on accurate diagnosis, intraoperative assessment, effective hemostasis, and adequate coronal sealing. However, given the limitations of a single case and relatively short follow-up period, these findings should be interpreted with caution, and further clinical evidence is required before generalizing outcomes.
